# Emotional distress in COVID-19 patients in Maldives

**DOI:** 10.1186/s12888-022-03826-1

**Published:** 2022-03-15

**Authors:** Rajib Kumar Dey, Shanooha Mansoor, Abdullah Isneen Hilmy, Sheena Moosa, Shiraany Abdul Rahman, Raishan Latheef, Nihla Rasheed, Fathimath Guraishaa Hassan, Ali Zaadhee, Afa Ibrahim, Sofoora Kawsar Usman

**Affiliations:** 1 Department of Internal Medicine, Indira Gandhi Memorial Hospital, PO:20402, Malé, Maldives; 2 Center for Mental Health, Indira Gandhi Memorial Hospital, PO:20402, Malé, Maldives; 3 Maldives National University, Malé, Maldives; 4 Department of Surgery, Indira Gandhi Memorial Hospital, PO:20402, Malé, Maldives; 5 National Cardiac Center, Indira Gandhi Memorial Hospital, PO:20402, Malé, Maldives; 6 Ministry of Health, PO: 20379, Malé, Maldives

**Keywords:** Anxiety, COVID-19, DASS21, Depression, Emotional distress, Pandemic, Stress

## Abstract

**Background and objectives:**

Researchers are exploring the epidemiology, clinical characteristics, treatment, vaccination and the challenges faced by healthcare authorities. However less focus is being paid towards the impact of COVID-19 on mental health of the patients. This study is a cross-sectional study, measuring the prevalence of emotional distress among patients with COVID-19 in the Maldivian population.

**Methods:**

This study was conducted in Maldivian nations above 18 of age with COVID-19 who were admitted in isolation facilities. Patients who were on treatment for any other chronic medical conditions, severe and critical COVID-19 disease were excluded. This study was conducted over a period of 2 months by administering a local translated version of DASS21 questionnaire.

**Results:**

The total of 195 patients were included in this study. The mean age of the patients was 40 (CI at 95% 38–42) years. The respondents were 48.7% men and 51.3% women. Overall, 9% of patients with COVID-19 had depression while 23% of patients had anxiety and 12% of the patients had stress. There was a statistically significant relationship between gender and depression, anxiety and stress (*p* < 0.01). Symptomatic cases had a significantly higher level of stress than asymptomatic patients (*p* < 0.05), but no significant association was observed with symptomatic status and anxiety or depression.

**Conclusion:**

The management of patients with COVID-19 should be multi-disciplinary with special focus on the mental wellbeing of our patients. We should aim to establish proper communication with the patients in order to identify emotional distress and provide appropriate mental health care.

**Supplementary Information:**

The online version contains supplementary material available at 10.1186/s12888-022-03826-1.

## Introduction

The Novel Coronavirus disease 2019 (COVID-19) pandemic outbreak that began in December 2019 in Wuhan China [1], rapidly spread throughout the world overwhelming the health systems in most of the countries. In the Maldives, as per Health Protection Agency, on December 3rd 2021, there were 91,993 confirmed cases with 251 deaths [2]. To help control the infection, governments have imposed strict lockdown at different occasions which has affected the social and mental wellbeing of the people. Mainstream and social media have been spreading awareness and constantly updating information regarding the rapid spread of the infection and dire outcome of the patients. COVID-19 positive patients are being isolated for long periods of time, some needing ventilatory support in the Intensive care unit while many high-risk patients eventually succumb to the disease. All these factors, collectively has led to a significant anxiety and fear among the people, especially amongst the patients who have contracted the virus. COVID-19 positive patients also go through stress as they are concerned not only of their fate, but also the fear of spreading the infection among family members and other close contacts [3].

Studies done during the Severe Acute Respiratory Syndrome (SARS) and Middle East Respiratory Syndrome (MERS) outbreaks showed that there were psychological disorders in the survivors [4–6]. Depression and post-traumatic stress disorder have been reported in these outbreaks even after 1 year of the illness [5]. As COVID-19 has been around for over 1 year there are different perspectives of mental health workers regarding the different emotional issues and related psychiatric disorders amongst the positive patients [7, 8]. .Studies have shown varying degree of psychiatric disorders during previous epidemics, which includes panic disorder, stress, depression, anxiety, post-traumatic stress disorder and acute psychosis. During SARS outbreak in 2003, Chua et al., reported a range of psychological responses to Perceived Stress Scale (PSS). Stress was significantly higher in SARS patients compared to the healthy control subjects with significantly negative psychological effects [5]. Laura Hawryluck et al. conducted a survey in 2004 after the SARS outbreak, where 129 quarantined persons who responded exhibited a high prevalence of psychological distress. Symptoms of posttraumatic stress disorder (PTSD) and depression were observed in 28.9 and 31.2% of respondents, respectively [4]. According to a global survey, there were various factors which contributed to the development of PTSD in quarantine facilities such as, different religious practices, forced quarantine, education level [9]. Jeong H, et al. conducted a study on the mental health status of people isolated due to MERS in 2016. Among 1656 patients isolated, anxiety symptoms were seen in in 7.6% and feelings of anger were present in 16.6% during the isolation period. At 4 to 6 months after release from isolation, anxiety symptoms were observed in 3.0% and feelings of anger were present in 6.4% [6]. Wang, et al. conducted a study in the general population in China to understand their levels of psychological impact, anxiety, depression, and stress during the initial stage of the COVID-19 outbreak. This study included 1210 respondents from 194 cities in China. In total, 53.8% of respondents rated the psychological impact of the outbreak as moderate or severe, 16.5% reported moderate to severe depressive symptoms, 28.8% reported moderate to severe anxiety symptoms, and 8.1% reported moderate to severe stress levels [10].

Researchers are exploring the epidemiology, clinical characteristics, treatment, vaccination and the challenges faced by healthcare authorities. Till date a total of 364,402 people have been fully vaccinated with two doses [2]. However less focus is being paid towards the impact of COVID-19 on mental health of the patients. Hospital data shows that the demand for mental health services was more than double during the pandemic, with 17,708 consultations provided by a multidisciplinary team of mental health professionals in 2020, in comparison to 7246 consultations in 2019. Therefore, we conducted a cross-sectional study, to measure the prevalence of emotional distress among patients with COVID-19 in the Maldivian population. This study will assist the healthcare professionals by providing them beneficial information that can be used to address the psychological wellbeing of the patients.

## Material & methods

This is a cross sectional study which was conducted in confirmed COVID-19 patients (i.e positive reverse transcriptase, polymerase chain reaction (RT-PCR) assay of nasal/oropharyngeal swabs) who were admitted in designated hospital-based isolation facilities.

All patients included in this study were Maldivian nationals, above 18 years of age. Written informed consent was taken from all the patients. Patients who were on treatment for any other chronic medical conditions like malignancy, cerebrovascular disease, chronic heart disease, chronic kidney disease and chronic liver disease or any other mental health disorders were excluded. Patients with severe and critical COVID-19 disease were also excluded from this study, as these patients were on high flow oxygen or ventilator.

This study was conducted from October 15th, 2020 to December 15th, 2020 by administering a local translated version (Dhivehi Language) of DASS21 questionnaire which was approved by the National Health Research Council of Maldives.

DASS 21 is a reliable tool used widely to assess the emotional distress in clinical and non-clinical population [11]. Psychometric properties of DASS 21 has been studied in adult cohorts of both genders in clinical and non-clinical samples with good internal consistency amongst the three subscales as well as the total score [12–14].

DASS 21 questionnaires were self-administered and those patients who had difficulty in filling the questioners were provided the required assistance by the research team. This questionnaire was provided on day seven of admission. Demographic data like Age, Sex, Marital Status and Disease severity were obtained from the patient’s medical records. For patient whose screening indicated a need for a psychosocial intervention, help was provided.

Analysis was be done after entering data into Stata. The main method for data analysis was Exploratory Factor Analysis along with regression, T-test and descriptive analysis. Continuous and categorical variables are expressed as mean (standard deviation (SD)) and frequency (percentage) respectively. T tests and regressions was used to investigate the association between qualitative variables with mental health status. A *P* value less than 0.05 was considered as statistically significant.

This research was approved by the National Health Research Council of the Maldives, on October 6th, 2020 (Research registration number NHRC/2020/015).

## Results

The total number of participants in the study was 195 patients and all of the questionnaires were filled completely (response rate 100%). The mean age of the patients was 40 (CI at 95% 38–42) years. The respondents were 48.7% men and 51.3% women. Out of the participants 72.3% were married, 22.6% were single, 2.6% were divorced and 2.7% were widowed. Therefore, the analysis considered married versus all other groups combined.

**Fig. 1:**
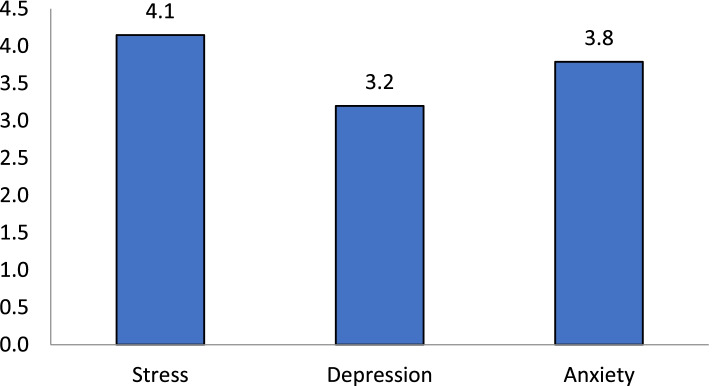
Mean of depression, anxiety and stress score in hospitalized patients with COVID-19

**Fig. 2:**
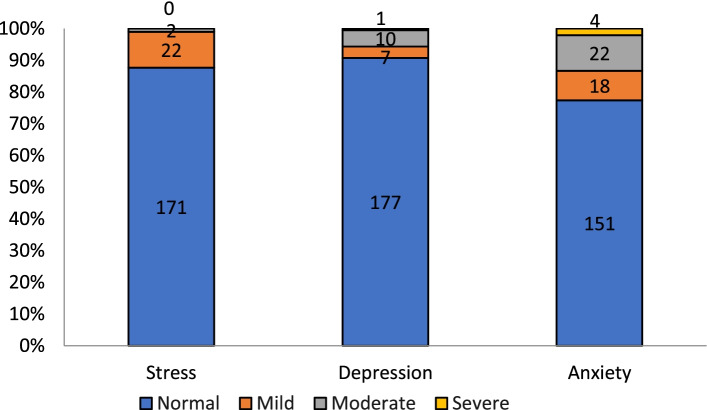
Prevalence of depression, anxiety and stress in hospitalized patients with COVID-19

Average subclass scores based on the overall score of the DASS-21 on depression was 3.2 (SD = 4.35), anxiety was 3.79 (SD = 4.38) and stress was 4.15 (SD = 4.83) (Fig. 1). Overall, 9% of patients with COVID-19 had depression while 23% of patients had anxiety and 12% of the patients had stress (Fig. 2).

There was no statistically significant association between the age of respondents with depression (*p* = 0.875), anxiety (*p* = 0.195) and stress (*p* = 0.291). Similarly, there was no statistically significant association between marital status or Covid-19 symptoms with depression, anxiety and stress (Table 1). However, there was a statistically significant relationship between gender and depression among patients admitted with COVID-19 (*p* < 0.01). There was also a significant association between gender and anxiety as well (*p* < 0.01). Similarly, for stress also there was a significant association (*p* < 0.01). This shows that females were more likely to experience mental health problems in relation to COVID-19 facility-based care. Symptomatic cases had a significantly higher level of stress than asymptomatic patients (*p* < 0.05), but no significant association was observed with symptomatic status and anxiety or depression.

**Table 1:** Demographic information of hospitalized patients with COVID-19 (*N* = 195)

	Depression					Anxiety					Stress				
	N	%	Mean	CL	***P***-value	N	%	Mean	CL	***P***-value	N	%	Mean	CL	***P***-value
**Male**	95	48.72	2.02	1.3–2.7		95	48.72	2.26	1.65–2.87		95	48.72	2.64	1.97–3.34	
**Female**	100	51.28	4.32	4.83–3.36	0.0002	100	51.28	5.24	4.25–6.23	0.0000	100	51.28	5.58	4.48–6.67	0.0000
**Unmarried**	54	27.69	3.37	1.93–4.81		54	27.69	3.89	2.68–5.08		54	27.69	4.17	2.86–5.48	
**Married**	144	72.31	3.13	2.78–3.79	0.7363	144	72.31	3.75	3.02–4.48	0.8454	144	72.31	4.14	3.33–4.5	0.9745
**Asymptomatic**	53	27.18	2.45	1.30–3.60		53	27.18	2.94	1.68–4.21		53	27.18	2.98	1.64–4.32	
**Symptomatic**	142	72.82	3.48	2.75–4.21	0.1437	142	72.82	4.11	3.39–4.81	0.0992	142	72.82	4.58	3.79–5.38	0.0390

## Discussion

The COVID-19 pandemic has led to a prolonged lockdown, social isolation and the concerns of one’s own health and that of loved ones. This could result in increased emotional distress in the society [15]. There are multiple factors contributing to anxiety and stress in COVID-19 patients. Genetics, female gender, education level, forced quarantine and location of quarantine were the most important contributary factors [16]. Stress can increase the levels of pro-inflammatory cytokines leading to increased susceptibility to infection [17]. There can also be pathological activation of monocytes/macrophages and lymphocytes leading to a cytokine storm which may lead to Acute Respiratory Distress Syndrome (ARDS) and increase in oxygen demand [18, 19]. In addition to this, anxiety can also be caused through an Interlukin (IL)-17 pathway [20, 21]. Other factors may be pre-existing health conditions like mental illness or medical conditions like diabetes, decreased physical activity and uncertainty of treatment towards a novel infection.

In our study we found that women are more liable to emotional distress in all categories which includes depression, anxiety and stress. Many studies show similar association, in which women having more depression, anxiety and stress compared to men [22–24].

The gender difference in emotional distress may be due to the added responsibilities such as work from home, taking care of children and other family members at home and homeschooling. Some women had to leave the workforce in order to take care of their children and other family members. This multitasking leads to inefficient selfcare among women which may be detrimental to their mental health. There may be a possibility that the pre-pandemic social stressors may have been exacerbated during the pandemic due to limited social contact and emotional support [25].

We also found that symptomatic patients were more stressed compared to the asymptomatic patients. There was no significant difference in Anxiety and Depression among patient with or without symptoms. The symptomatic patients would have been more stressed as they were unaware and uncertain of the disease outcome in themselves and their loved ones, given the number of mortalities reported locally and worldwide. Although locally we had few cases in the Maldives, the information regarding high mortality rates in other countries like Italy and United States, were broadcasted in the mass media.

The findings from our study suggests that besides the medical management of COVID-19, we should also focus on the mental wellbeing of our patients. We should aim to establish proper communication with the patients in order to identify emotional distress and provide appropriate mental health care. There have been several resources developed to help patients address their mental well-being. These patients were followed up in the Psychiatry and Internal Medicine outpatient department and were offered counselling along with mental and physical rehabilitation. Due to the lack of mental health services in primary care, and a dearth of mental health professionals working in different regions of the country, mental health services are primarily provided through the central government hospital located in the capital city in Maldives. Mental health services are available in a few private clinics, however there is a lack of national data and prominent research on mental health in the country.

Apart from the general techniques that can be taught to patients like breathing, maintain a routine, maintaining contact as suggested by CDC and WHO, the environment could be made more conducible [26]. Like the setting, having an open area for walking, electronic medium, means to contact family and offering social support [27].

## Conclusion

The management of patients with COVID-19 should be multi-disciplinary with special focus on the mental wellbeing of our patients. We should aim to establish proper communication with the patients in order to identify emotional distress and provide appropriate mental health care. The frontline staff should be provided additional training, starting from how to break bad news, how to have a compassionate conversation with those at high risk and who are critically unwell. This will help to identify what are important to patient and hence can be incorporated to improve quality of care. Identifying those needing additional support, establishing a referral pathway and incorporating guidance about stress into general care practices are important according to American Medical Association [28].

## Limitations

The number of moderate cases in this study is considerably less and also, we have excluded all severe cases in whom the emotional distress may have been more.

Other demographic factors such as education and occupation has not been considered in analysis of emotional distress in the patients.

## Supplementary Information

**Additional file 1.**

## Data Availability

Raw data is available in the supplementary material.
